# Associations of non-HDL-C and triglyceride/HDL-C ratio with coronary plaque burden and plaque characteristics in young adults

**DOI:** 10.17305/bjbms.2022.7142

**Published:** 2022-05-08

**Authors:** Fatih Akin, İbrahim Altun, Burak Ayca, Nuri Kose, İlknur Altun

**Affiliations:** 1 Department of Cardiology, Muğla Sıtkı Kocman University School of Medicine, Muğla, Turkey; 2 Department of Cardiology, İstanbul Education and Research Hospital, Istanbul, Turkey; 3 Department of Cardiology, Muğla Yucelen Hospital, Muğla, Turkey; 4 Department of Radiology, Muğla Sıtkı Kocman University School of Medicine, Muğla, Turkey

**Keywords:** Non-high-density lipoprotein cholesterol, triglyceride/high-density lipoprotein cholesterol ratio, coronary plaque characteristic, coronary computed tomography angiography

## Abstract

Coronary artery disease (CAD) is uncommon in young adult patients. However, these patients have different risk factor profiles and high-risk coronary plaques are more common. The aim of this study was to examine the relations between the coronary plaque burden, plaque composition, serum non-high-density lipoprotein cholesterol (non-HDL-C) levels, and triglyceride/high-density lipoprotein cholesterol (TG/HDL-C) ratio in young adults. We analyzed a total of 551 patients under age 45 who had undergone coronary computed tomography angiography (CCTA). Coronary plaque characteristics were analyzed using CCTA. Multivariate linear regression analysis was used to assess the predictors of non-calcified plaque burden (NCB) and calcified plaque burden (CB) burdens. Serum non-HDL-C levels and TG/HDL-C ratio were higher in the coronary atherosclerosis patient group. Serum non-HDL-C levels and the TG/HDL-C ratio were higher in the obstructive CAD patient group. The plaque burden was positively correlated with non-HDL-C (r = 0.30; *p* < 0.001) and TG/HDL-C ratio (r = 0.18; *p* < 0.001). NCB was positively correlated with age, gender, smoking status, fasting blood glucose, total cholesterol, low-density lipoprotein cholesterol, serum triglycerides, HbA1c, non-HDL-C, and TG/HDL-C ratio. Non-HDL-C (β coefficient = 0.13; *p* = 0.023) and TG/HDL-C ratio (β = 0.10; *p* = 0.042) were independent predictors of NCB. Serum non-HDL-C levels and TG/HDL-C were significantly associated with the presence and burden of coronary plaques. Serum non-HDL-C and TG/HDL-C ratios were independently associated with NCB, suggesting their use as easy-to-compute markers for identifying high-risk groups in young adults.

## INTRODUCTION

Abnormalities in serum lipids, including elevated blood low-density lipoprotein cholesterol (LDL-C), decreased high-density lipoprotein cholesterol (HDL-C), and elevated blood triglyceride (TG) levels, are well-known risk factors for coronary atherosclerosis (CA) [1,2]. While LDL-C is considered the most important lipoprotein risk factor, some recent epidemiologic studies have suggested that non-HDL-C may be superior to LDL-C for identifying the risk of coronary artery disease (CAD) [3,4].

Non-HDL-C is easily calculated from a lipid profile (non-HDL-C = total cholesterol (TC) minus HDL-C) and represents all apolipoprotein B (apoB)-containing lipoproteins (including LDL-C), very low-density lipoproteins and their metabolic remnants, intermediate-density lipoproteins, and lipoprotein(a) [5]. Recent European and United States guidelines recommend LDL-C as a primary cardiovascular disease (CVD) risk indicator and non-HDL-C as a secondary CVD risk indicator [6,7]. The TG/high-density lipoprotein cholesterol (TG/HDL-C) ratio, known as an atherogenic index of plasma, provides additional risk stratification beyond the one provided by LDL-C [8]. Although the relation between TG/HDL-C and several CVDs is well known, its relation with coronary plaque morphology remains unclear.

Coronary computed tomography angiography (CCTA) allows accurate evaluation of CA, including lesion location and severity, and provides further information about plaque characteristics [9]. Non-calcified plaque (NCP) detected by CCTA has been linked with higher risk of CVD in patients with severe hypercholesterolemia [10]. Results from the multicenter SCOT-HEART trial showed that non-calcified low-attenuation plaques, which are more vulnerable than calcified plaques (CP), seen on CCTA might predict increased coronary events better [11]. Young adults display high-risk coronary plaque characteristics more frequently than is observed in older adults [12]. Although current guidelines recommend serum cholesterol monitoring in young adults, few patients were monitored for dyslipidemia in the early adulthood. Nevertheless, evaluation of lipid disorders in the early adulthood is important for implementation of effective management strategies, such as lifestyle modification or medications. The associations between coronary plaque characteristics, non-HDL-C levels, and TG/HDL-C ratios have also not been studied previously. The aim of the present study was therefore to determine the associations of non-HDL-C and the TG/HDL-C ratio with the coronary plaque burden and characteristics detected by CCTA in adults young adults.

## MATERIALS AND METHODS

### Study population

Patients who underwent CCTA between March 2018 and January 2020 were considered for possible inclusion in the study. Patients admitted or referred to our cardiology polyclinics with chest pain or any symptoms related to stable CAD were included in the study. Other criteria included the evaluation of asymptomatic patients who had intermediate to high-risk scores, according to the Framingham risk assessment. Over 2000 CCTA examinations were performed annually in our center; however, our main inclusion criterion was to select patients younger than 45 years of age. A total of 258 consecutive patients with CAD and 293 patients with normal coronary arteries (control group) were included in the present study using the following exclusion criteria: Patients with the previous coronary artery bypass grafting, percutaneous transluminal coronary angioplasty, acute coronary syndrome (ACS), concomitant inflammatory diseases, or neoplastic diseases.

Patients taking lipid-lowering agents were also excluded from the study. CCTA was commonly employed due to clinical symptoms or for evaluation of asymptomatic patients who had intermediate to high Framingham risk scores. Hypertension was defined as antihypertensive drug use. The DM was defined as fasting blood glucose >126 mg/dL or being on treatment. The current smokers were defined as having a history of smoking within the past year. The study protocol was reviewed and approved by an Internal Ethical Review Board (reference number 200021).

### Image analysis

All scans were interpreted using the three-dimensional Syngo.via workstation (dual-source CT, Somatom Definition Flash, Siemens Healthcare, Erlangen, Germany) by a cardiologist and radiologist, both level 3 certified, who were blinded to the individual’s clinical findings. The final CCTA diagnosis was determined by consensus interpretation. The identification of plaque and stenosis, as well as the amount of plaque per segment, was determined by the highly trained cardiologist and radiologist, as proposed by the modified American Heart Association classification [13].

The coronary artery calcium (CAC) score was measured with the Agatston method [14]. The Agatston score was computed as the integral (sum) of all Hounsfield values in a lesion multiplied by the voxel volume in mm^3^. Plaques were defined as structures in the artery that was within 1 mm^2^ of the vessel lumen or directly adjacent and that could be clearly distinguished from the surrounding pericardial tissue and the vessel lumen. CP consisted of more than 50% calcified tissue (density C130 HU in native scans), mixed plaque (MP) contained <50% calcified tissue, and plaques lacking signs of calcification which were categorized as NCP [15].

Plaque burden, calcified plaque burden (CB), and non-calcified plaque burden (NCB) were measured by summing the number of coronary artery segments that possessed each respective plaque type. CA was defined as the presence of plaque during the CCTA examination. Non-obstructive coronary atherosclerosis (NOCA) was defined as the presence of <50% stenotic plaque during the CCTA examination. Obstructive CAD was defined as the presence of 50% or more stenotic plaque during the CCTA examination.

### Laboratory measurements

Venous blood was collected from all study subjects after an overnight fast (from 8:00 p.m. to 9:00 a.m.). Enzymatic colorimetric assays were used to measure TC and TG levels, and serum HDL-C was measured using a homogeneous enzymatic colorimetric test. The Friedenwald equation was used to calculate LDL-C levels when the TG level was ≤400 mg/dL. Serum non-HDL-C and TG/HDL-C ratio levels were calculated by subtracting the HDL-C level from the TC level and by dividing TG by the HDL-C value, respectively. An immunoturbidimetric method was used to determine the high-sensitivity C-reactive protein (hs-CRP) concentration [16]. Fasting plasma glucose, TG, TC, LDL-C, HDL-C, creatinine, and hemoglobin A1c (HbA1c) were determined using standardized methods.

### Statistical analysis

All analyses were performed using SPSS 20.0 (released 2011, IBM statistics for Windows version 20, IBM Corp., Armonk, NY). Comparison of parametric values between the two groups was performed with an independent samples t-test. Comparisons of nonparametric values between the two groups were performed with the Mann–Whitney U- test. Categorical variables were compared by the Chi-square test. The point-biserial correlation is commonly used to measure the strength and direction of the relationship that exists between one continuous variable and one binary variable. We used point-biserial correlation to test correlations between one continuous variable and one dichotomous variable [17].

Pearson correlation coefficient was computed to examine the association between two continuous variables. Linear regression analysis was used to assess the predictors of the CB and NCB. All variables associated with these parameters with a level of significance of <0.1 were included in the tested model including Framingham risk factors and emerging risk factors. Variables with *p* < 0.1 determined by univariate analysis were included in the backward step-wise multivariate regression analysis model. A two-tailed *p* < 0.05 was considered statistically significant.

## RESULTS

### Study population characteristics

The clinical and demographic properties of the study population are presented in Table 1. Patients with CA were older and had a higher prevalence of male gender, hypertension, diabetes mellitus, and tobacco use. Similarly, serum fasting blood glucose, TC, TG, LDL-C, and HbA1c levels were higher in the patients with CA than in patients without CA, whereas serum HDL-C was lower in the patients with CA. Both the serum non-HDL-C levels and the TG/HDL-C ratios were more elevated in the CA group of patients (169.6 ± 46 mg/dL vs. 142.1 ± 48 mg/dL and 5.9 ± 4.6 vs. 3.8 ± 3.7, respectively; *p* < 0.001 for both). However, serum hemoglobin, white blood cell (WBC) counts, and hs-CRP were not significantly different.

**TABLE 1:**
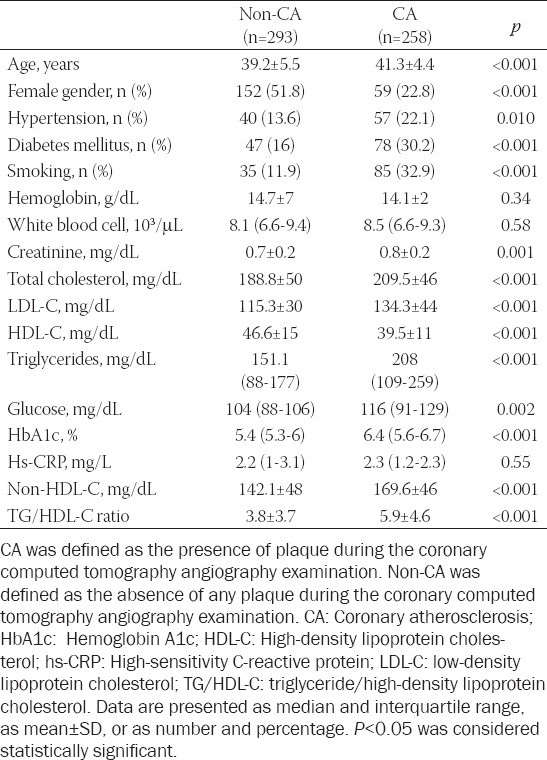
Characteristics of the study population

### Population characteristics of the CA subgroup

**FIGURE 1:**
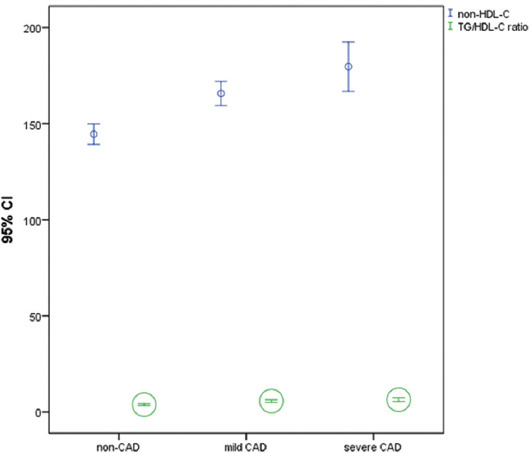
Graph shows comparison of non-high-density lipoprotein cholesterol (non-HDL-C), triglyceride/high-density lipoprotein cholesterol (TG/HDL-C) ratio between non-coronary artery disease (CAD), non-obstructive coronary atherosclerosis (NOCA) and obstructive CAD groups in young adults. P <0.05 was considered statistically significant.

We divided the patients with CA into two subgroups according to their CA severity. Among the 258 participants, 70 patients had obstructive CAD (≥50% stenotic plaque) and 188 had NOCA (<50% stenotic plaque). Patients with obstructive CAD commonly had a history of hypertension and diabetes mellitus. Serum fasting blood glucose, TC, TG, LDL-C, HbA1c, WBC, and hs-CRP values were higher in the patients with obstructive CAD than with NOCA, whereas HDL-C and creatinine values were not significantly different. Both serum non-HDL-C levels and TG/HDL-C ratio were also more elevated in the patients with obstructive CAD than with NOCA (179.6 ± 53 mg/dL vs. 165.8±43 mg/dL and 6.3 ± 4.1 vs. 5.7 ± 4.8, respectively; *p* = 0.036 vs. *p* = 0.045, respectively) (Figure 1). The CB, NCB, and Agatston score values were also higher in the patients with obstructive CAD than with NOCA (Table 2).

**TABLE 2:**
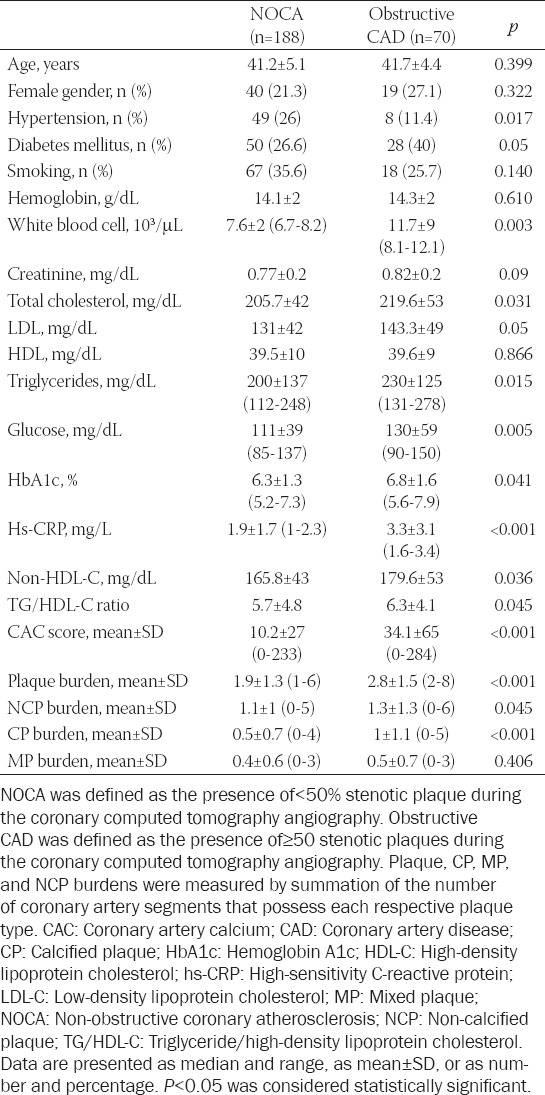
Characteristics of the population according to CAD severity

### Association between non-HDL-C levels and TG/HDL-C and different parameters

**FIGURE 2:**
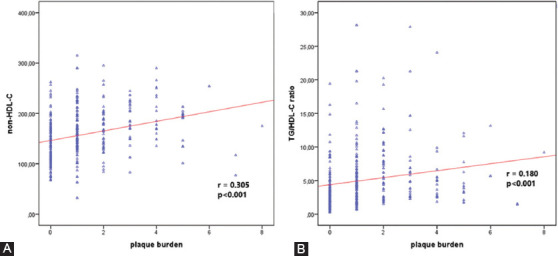
Graphs show significant correlations between the plaque burden and (A) non-high-density lipoprotein cholesterol (non-HDL-C); and (B) triglyceride/high-density lipoprotein cholesterol (TG/HDL-C) ratio in young adults. p < 0.05 was considered statistically significant.

The plaque burden was positively correlated with non-HDL-C (r = 0.30; *p* < 0.001), and TG/HDL-C ratio (r = 0.18; *p* < 0.001) (Figure 2A and B). The CB was positively correlated with age (r = 0.25; *p* < 0.001), hypertension (β = 0.145; *p* = 0.003), TC (r = 0.20; *p* < 0.001), LDL-C (r = 0.18; *p* < 0.001), and non-HDL-C (r = 0.22; *p* < 0.001) and was negatively correlated with HDL-C (r = −0.11; *p* = 0.022). No association was detected with gender, serum creatinine, TG, WBC, or hs-CRP.

**FIGURE 3:**
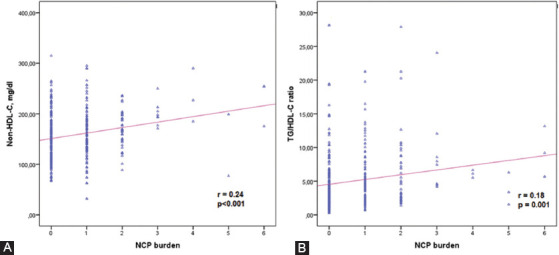
Graphs show significant correlations between non-calcified plaque (NCP) burden and (A) non-high-density lipoprotein cholesterol (non-HDL-C); (B) triglyceride/high-density lipoprotein cholesterol (TG/HDL-C) ratio in young adults. p < 0.05 was considered statistically significant.

The NCB was positively correlated with age (r = 0.13; *p* = 0.006), gender (β = 0.120; *p* = 0.016), smoking status (β = 0.223; *p* < 0.001), fasting blood glucose (r = 0.15; *p* = 0.005), TC (r = 0.19; *p* < 0.001), LDL-C (r = 0.20; *p* < 0.001), serum TG (r = 0.15; *p* = 0.003), HbA1c (r = 0.28; *p* < 0.001), non-HDL-C (r = 0.24; *p* < 0.001), and TG/HDL-C ratio (r = 0.18; *p* = 0.001) and was negatively correlated with HDL-C (r = −0.23; *p* < 0.001) (Table 3). The significant relationship between the NCB and the serum non-HDL-C and TG/HDL-C ratio is shown in Figure 3A and B.

**TABLE 3:**
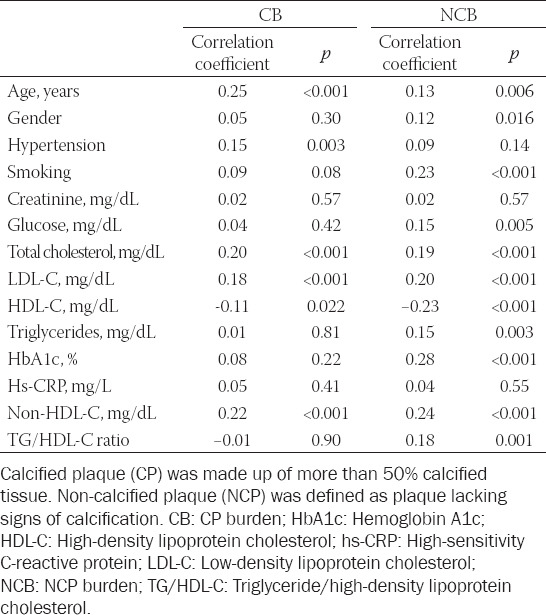
Correlations between CB and NCB and different variables evaluated in the study

### Predictors of CP and NCP burden

To further analyze the independent contribution of non-HDL-C to the variance of CB and NCB, we used linear regression analysis based on traditional and non-traditional risk factors impacting on this variable. Age (β = 0.17; *p* = 0.001), hypertension (β = 0.11; *p* = 0.037), and non-HDL-C (β = 0.14; *p* = 0.011) were independent indicators of CB. Smoking (β = 0.19; *p* = 0.001), fasting blood glucose (β = 0.15; *p* = 0.002), and non-HDL-C (β = 0.12; *p* = 0.024) were independent predictors of the NCB (Table 4).

**TABLE 4:**
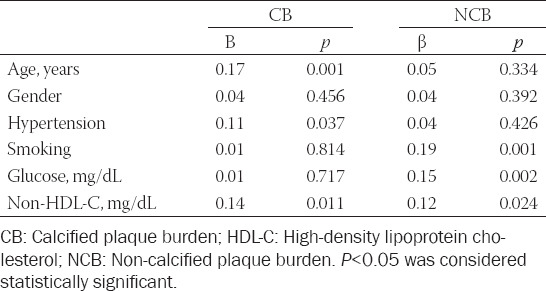
Associations of serum non-HDL-C and risk factors for CAD with CB and NCB

Associations of serum TG/HDL-C ratio and risk factors for CAD with CB and NCB are shown in Table 5. Age (β = 0.14; *p* < 0.001) and hypertension (β = 0.10; *p* = 0.038) were independent indicators of CB. Smoking (β = 0.19; *p* < 0.001), fasting blood glucose (β = 0.14; *p* = 0.004), and TG/HDL-C ratio (β = 0.10; *p* = 0.042) were independent predictors of the NCB.

**TABLE 5:**
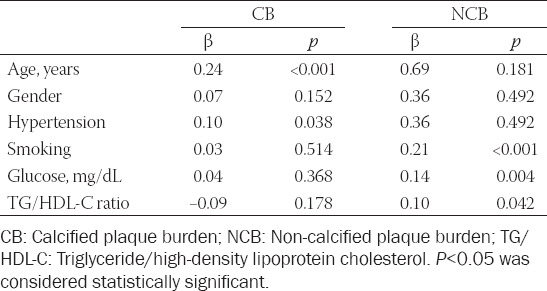
Associations of Serum TG/HDL-C ratio and risk factors for CAD with CB and NCB

## DISCUSSION

In our study, we found that serum non-HDL-C and the TG/HDL-C ratio were significantly higher in patients with CA than without CA, as detected by CCTA. Serum non-HDL-C and the TG/HDL-C ratio were also significantly associated with coronary plaque burden. Serum non-HDL-C and TG/HDL-C ratio were significantly associated with the NCB detected by CCTA in adults younger than 45 years.

NCB detected by CCTA was linked to large plaque lipid cores and may predict future ACS [18]. Consistent with our findings, Nakazato et al. [19] found a significant association between non-HDL C but not LDL-C and the NCB detected by CCTA. Our study confirms their findings and extends them to adults younger than age 45. Consistently, the results of cardiac studies have shown a significant relation between apoB in young adults (mean age: 25 years) and midlife CAC (mean age: 50 years), suggesting that apoB may independently predict future CAD in young adults [20]. Another report indicated that non-HDL-C ahead of LDL-C may predict the severity of CAD detected by coronary angiography [21]. However, Kurmus et al. [22] found no significant association between non-HDL-C and CAD severity detected by coronary angiography.

A direct link exists between the number of apoB-containing lipoproteins and the level of non-HDL-C. ApoB-carrying lipoproteins affect the atherosclerotic process by accumulating within the arterial wall [23,24]. Non-HDL-C is also correlated with the LDL-particle number, which may predict future CVD better than LDL [25,26]. Results from the Multinational Cardiovascular (CV) Risk Consortium suggest that non-HDL-C may predict long­term CV risk [27]. In this study, patients younger than 45 years showed the strongest HRs for the relation between non­HDL-C and long-term CV events, whereas a weaker association was found for non­HDL-C with the incidence of CVD during long-term follow-up in older individuals (>60 years). Most adults with increased non­HDL-C blood concentrations in the early adulthood continue to have high lifetime non-HDL-C and a higher risk of CAD. Non-HDL-C levels in young adults are generally stable over their life course; therefore, early detection of cholesterol abnormalities, earlier lifestyle changes, and further follow-up is important for reducing their long-term risk of CVD [28].

Recent studies comparing the predictive value of LDL-C versus non-HDL-C have suggested that non-HDL-C may have more predictive ability than LDL-C for future CV events [3,29]. Therefore, in addition to reducing the LDL-C levels, lowering non-HDL-C and TG may further reduce the risk of CA, especially early in life. In line with this assumption, our study suggests that earlier lifestyle changes and lipid-lowering pharmacologic interventions may yield greater clinical benefits than are achievable with later lifestyle and pharmacologic interventions.

Decreased lipoprotein lipase activity and increased levels of serum small dense LDL-particle are associated with lower HDL-C levels and higher TG levels [30]. Thus, the TG/HDL-C ratio may represent an easy-to-measure statistic for screening for TG metabolism abnormalities. TG/HDL-C ratio as a combination of two dyslipidemia parameters have been found to be positively correlated with insulin resistance [31]. In line with this assumption, emerging data are linking TG/HDL-C ratio to diabetes and metabolic syndrome [32,33]. Furthermore, the previous studies demonstrated that TG/HDL-C ratio may be a predictor of CV death, and all-cause mortality [34]. TG/HDL-C ratio has been found to be associated with increased risk of major CV adverse events in women with NOCA [35]. Furthermore, it was reported that TG/HDL-C ratio may predict development of future CVD in healthy non-diabetic middle-aged men [36].

The TG/HDL-C ratio was previously identified as a possible predictor of the development of future CVD in healthy non-diabetic middle-aged men [36]. Dogan et al. [37] also investigated the association between the TG/HDL-C ratio and the risk of acute myocardial infarction (AMI) in the early adulthood. In their study, the TG/HDL-C ratio was significantly higher in younger AMI patients, whereas LDL-C levels were similar in both younger and older patients. Their study findings suggested that the TG/HDL-C ratio may have a greater predictive ability than LDL-C for future AMI and that the predictive ability of the TG/HDL-C ratio may differ with age. A recent study by Chen and Dai [38] showed a significant association between TG/HDL-C ratio and arterial stiffness detected by brachial-ankle pulse wave velocity in Japanese population. In our study, the TG/HDL-C ratio was an independent predictor of the NCB. In our study, the hs CRP level did not differ between the CA and non-CA groups, but it was quite different between the “NOCA” and “obstructive” CAD groups. Young population of our study and exclusion of patients with ACS could be the reasons for the lack of an association between the hs-CRP and CA groups.

Our study included only individuals under 45 years of age from Turkey; therefore, the generalizability of our findings to other ages and regions is unknown. We also excluded patients taking lipid-lowering therapy; therefore, the therapeutic effect of lipid-lowering therapy was not investigated in our study. We also did not measure lipoprotein subfractions (including apolipoprotein A and apoB levels), and measurement of these lipoprotein subfractions could provide additional information about the underlying mechanisms. Information on body mass index before the CCTA examination was not available for some of our patients; therefore, we could not include body mass index in our analysis. Nevertheless, this is the first study that demonstrates an association between coronary plaque composition, serum non-HDL-C, and the TG/HDL-C ratio in young adults and utilizes CCTA with an adequate detection of coronary vessels and plaque morphology analyzed by a level 3 certified cardiologist and a level 3 certified radiologist. While previous studies have used arterial stiffness and the Agatston score to define subclinical atherosclerosis, we used CCTA with a sufficient number of patients.

## CONCLUSION

The non-HDL-C level and the TG/HDL-C ratio were significantly associated with the presence and burden of coronary plaques. Both the serum non-HDL-C level and the TG/HDL-C ratio were also correlated with the NCP morphology. Furthermore, non-HDL-C and TG/HDL-C ratio were independent predictors of the NCB and could be used as inexpensive and easy-to-measure statistics for determination of high-risk coronary plaques in young adults. Future studies investigating the association of non-HDL-C and TG/HDL-C with CV morbidity and mortality are needed.
